# Analysis of Risk Factors for Carbapenem-Resistant Klebsiella pneumoniae Infection and Its Effect on the Outcome of Early Infection After Kidney Transplantation

**DOI:** 10.3389/fcimb.2021.726282

**Published:** 2021-10-08

**Authors:** Fei Zhang, Jinbiao Zhong, Handong Ding, Jiashan Pan, Jing Yang, Tianchi Lan, Yiding Chen, Guiyi Liao

**Affiliations:** ^1^ Department of Urology, The First Affiliated Hospital of Anhui Medical University, HeFei, China; ^2^ Institute of Urology, The First Affiliated Hospital of Anhui Medical University, HeFei, China; ^3^ Anhui Province Key Laboratory of Genitourinary Diseases, Anhui Medical University, HeFei, China

**Keywords:** kidney transplant, carbapenem-resistant Klebsiella pneumoniae, early infections, risk factors, multidrug resistance

## Abstract

**Background:**

Infections remain a major cause of morbidity and mortality in kidney transplant (KT) recipients. This study was performed to identify the overall prevalence of early infections, prevalence of carbapenem-resistant Klebsiella pneumoniae (CRKP) infection after KT, one-year postoperative mortality in patients with early infections and risk factors for CRKP infections.

**Methods:**

We conducted a retrospective study of all patients who received KT in our hospital between January 2017 and December 2019. We evaluated the demographic, clinical, infection characteristics and the one-year postoperative outcomes.

**Results:**

Among the 419 patients who received KT between January 2017 and December 2019, 150 patients had at least one infection within 90 days after KT. The total prevalence of early infections was 36.1% (150/415), the prevalence of early CRKP infections was 10.4% (43/415), and the one-year postoperative mortality was 15.3% (23/150) in patients with early infections. The risk factors independently related to one-year postoperative mortality were mechanical ventilation (MV) > 48 h (Odds ratio (OR)= 13.879, 95%Confidence interval (CI): 2.265~85.035; P=0.004) and CRKP infection (OR=6.751, 95% CI: 1.051~43.369; P =0.044). MV> 48 h was independently related to CRKP infection (OR=3.719, 95% CI: 1.024~13.504; P=0.046). Kaplan-Meier survival curves showed that the one-year survival rate of patients infected with CRKP in the early postoperative stage was significantly lower than that of uninfected patients.

**Conclusions:**

In general, the prevalence of early infections after KT is high, and CRKP infection is closely correlated with poor prognosis. The effective prevention and treatment of CRKP infection is an important way to improve the one-year survival rate after KT.

## Introduction

Solid organ transplant (SOT) recipients have a higher risk of infection than other populations, mainly due to the following reasons: transplantation from marginal donors, especially infected donors; surgical intervention; and the use of immunosuppressants (Linares et al., 2010). Infection is a major cause of the high morbidity and mortality observed in KT recipients (Kim, 2014). Bacterial infection is the most common type of infection after transplantation, followed by fungal, viral and protozoal infections (Blair and Kusne, 2005). The vast majority of bacterial infections, most of which are caused by microorganisms in hospitals, occur in the first three months after transplantation (Fishman, 2011).

In recent years, the rates of infection with gram-negative bacteria and multidrug-resistant gram-negative bacteria (MDR-GNB) after SOT have increased. It is estimated that 10%-20% of SOT recipients are infected with MDR-GNB (Zhong et al., 2012; Bodro et al., 2013; Ye et al., 2014) of which carbapenem-resistant Klebsiella pneumoniae (CRKP) is the most lethal. In CRKP-endemic areas, the mortality rate of SOT recipients infected with CRKP was estimated to be close to 40%, which is approximately 3-5 times higher than that of non-CRKP-infected recipients (Kalpoe et al., 2012; Clancy et al., 2013; Pouch et al., 2015). These data indicate that early infection after transplantation, especially with CRKP, is related to a poor prognosis of the recipient.

This study was performed to identify the overall prevalence of early infections after KT and the risk factors for one-year postoperative mortality in patients with early infections. We also analyzed the prevalence of early postoperative CRKP infection and its risk factors.

## Materials And Methods

### Study Design and Patient Sample

This was a retrospective observational study conducted between January 2017 and December 2019, at the First Affiliated Hospital of Anhui Medical University, a tertiary teaching hospital with 2800 beds located in Anhui Province. The primary endpoint of our study was 1-year mortality of patients with early infections post KT, the secondary endpoint was prevalence of CRKP infection within 3 months post KT. The patient consisted of subjects who received KT in our hospital and survived more than 48 hours after transplantation, Patients infected with bacterial and fungal within three months after KT were included in our study. Among these infected patients, they were further divided into two subgroups, patients with early CRKP infections and patients without CRKP infections. Three recipients were excluded due to loss of follow-up data and one died within 48 hours, finally a total of 415 recipients were included. All recipients were regularly followed up in the outpatient department after transplantation. All the selected recipients received triple immunosuppression (tacrolimus or cyclosporin A, prednisone, and mycophenolate mofetil), and some received additional anti-thymocyte immunoglobulin. A third-generation cephalosporin/β-lactamase inhibitor or semisynthetic penicillin/β-lactamase inhibitor was used 1 hour before transplantation, and the same drug was used for at least 7 days after transplantation. This study was approved by our institutional Ethics Review Committee and was conducted in accordance with the principles of the Declaration of Helsinki.

### Clinical Data Collection

The patients’ clinical data, including demographics, infection characteristics, and preoperative, intraoperative, and postoperative variables, were reviewed by electronic medical records. The total prevalence of postoperative infections, the prevalence of CRKP infections since the day of transplantation and the one-year postoperative mortality rate were calculated. In the analysis of the risk factors for one-year mortality in the patients with early infections, we evaluated age, sex, donor type, diabetes mellitus, etiology of renal failure, acute rejection, site of infection, delayed graft function (DGF), anti-thymocyte globulin induction, mechanical ventilation (MV) > 48 h, multiple infections, fungal infection, cytomegalovirus (CMV) infection, multifocal infections, mixed infections, multidrug-resistant (MDR) bacterial infection, CRKP infection, postoperative leukopenia, reintervention and the length of ICU(intensive care unit) stay.

For the patients with early CRKP infections, only the first CRKP infection after transplantation were included in the study. We collected patient age, sex, diabetes mellitus, etiology of renal failure, donor type, acute rejection, CMV infection, DGF, anti-thymocyte globulin induction, postoperative leukopenia, reintervention, the length of ICU stay and MV> 48 h and assessed them as potential risk factors for CRKP infection.

### Definition

Early infection was defined as an infection occurring in the first three months after transplantation. The standards used to define and classify infections in our study were those proposed by the Centers for Disease Control and Prevention (Horan et al., 2008). In particular, factors such as pneumonia (including ventilator-associated infections), surgical site infections (SSIs), bloodstream infections (BSIs) (including catheter-associated infections) and urinary tract infections (UTIs) were taken into account. On the basis of clinical suspicion, the culture of bacteria or fungi from blood, sputum (or other respiratory secretions), urine or ascites samples was carried out for diagnostic purposes, and the occurrence of infection was defined based on the combination of a positive culture with clinical manifestations. CRKP was defined as insensitivity to at least one of the carbapenems, with a minimum inhibitory concentration ≥2 mg/mL for ertapenem and ≥4 mg/mL for imipenem or meropenem (Clinical and Laboratory Standards Institute, 2017). Reintervention was defined as any postoperative invasive procedure that required local or general anesthesia. DGF was defined as a decrease in daily serum creatinine less than 10% from the previous day for 3 consecutive days in the first postoperative week or serum creatinine failing to decrease to 400 μmol/L in the first postoperative week (Paloyo et al., 2016). Leukopenia was defined as a leukocyte count less than 3000 cells/μL detected at least once on a white blood cell test (Hamel et al., 2019).

### Microbiology

When any infection was suspected during the follow-up period, clinical samples were collected for culture, and microbial culture and identification were carried out in accordance with standard bacteriological procedures. Bacteria were cultured and identified with the VITEK-2 system (Biomerieux, Marcy-l ‘Etoile, France). The minimum inhibitory concentration was interpreted according to the breakpoint set by the Clinical and Laboratory Standards Institute (2017).

### Statistical Analysis

The statistical analysis was performed using SPSS software [Version 25.0; SPSS Inc., Chicago, IL, USA]. Categorical variables are described as frequencies and percentages. Continuous variables with normal distributions are described as the means and standard deviations; otherwise, they are described as the medians (IQRs). The Kolmogorov–Smirnov test was conducted to evaluate the normality of variable distribution. An independent-samples t-test was conducted to compare the means of two normally distributed variables. The Mann–Whitney U-test was conducted to evaluate nonnormally distributed variables. The X2 test was conducted to evaluate the difference between categorical variables. The risk factors for one-year mortality in patients with early infections after KT were identified by univariate and multivariate logistic regression analyses using odds ratios (ORs) and 95% confidence intervals (CIs). Univariate and multivariate logistic regression analyses were also carried out to identify the risk factors for CRKP infection. Variables that are significant at the univariate level (P<0.05) were included in the stepwise binary Logistic regression model to analyze the factors affecting the outcome. The 10-fold cross validation method is used to verify the robustness of the model. Kaplan-Meier analysis and the log-rank test were conducted to evaluate the difference in the one-year survival curves between patients with and without CRKP infections. P<0.05 was considered statistically significant.

## Results

According to the medical records, 419 patients received KT between January 2017 and December 2019, a total of 419 patients received kidney transplantation, three recipients with lost follow-up data and one recipient who died within 48 hours after KT were excluded, 150 of the 415 included recipients recorded at least one infection within 90 days after transplantation. The total prevalence of early infections was 36.1%, and the prevalence of MDR bacterial infections was 22.9%. A total of 69.3% of the 150 patients received kidneys donated by deceased donors. The most common etiology of renal failure was glomerulonephritis.

### Infection Site and Microbial Etiology

**Figure 1:**
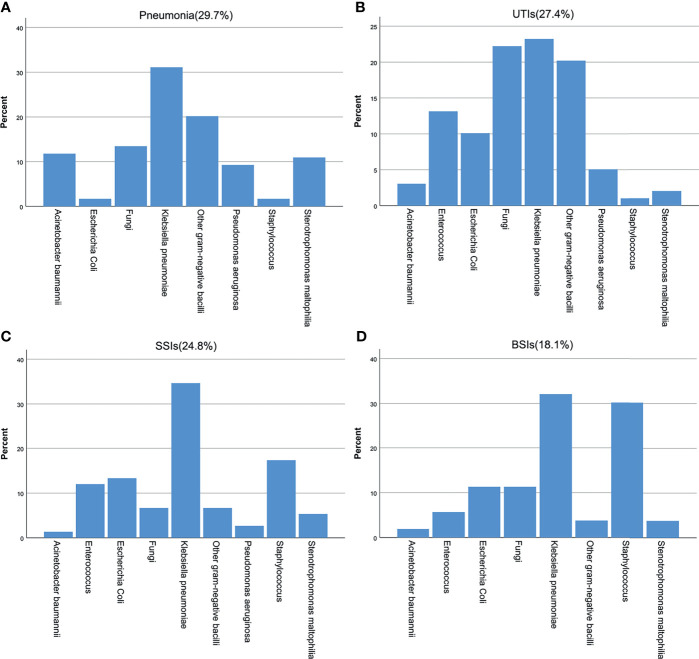
Pathogen distribution at different infected sites after KT. **(A)** pneumonia, **(B)** UTIs, **(C)** SSIs, **(D)** BSIs.

Of the 150 patients, 95 patients (63.3%) had infections at only one site, and 55 patients (36.7%) had infections at ≥ 2 sites. Of the 226 recorded infections, 67 (29.7%) were pneumonia, 62 (27.4%) were UTIs, 56 (24.8%) were SSIs, and 41 (18.1%) were BSIs. A total of 347 pathogens were isolated from the sites of infection, of which 69.5% were gram-negative bacteria, 16.4% were gram-positive bacteria and 14.1% were fungi. K. pneumoniae (42.7%) was the most common pathogen among the gram-negative bacteria, followed by Escherichia coli (10.4%) and Stenotrophomonas maltophilia (8.7%). Among the gram-positive bacteria, Enterococcus faecium (28%) was the most common, followed by Staphylococcus epidermidis (19.3%) and Staphylococcus aureus (14.0%). Among the fungi, C. glabrata (38.8%) was the most common. The distribution of bacteria at different infection sites is shown in **Figure 1**.

In terms of drug resistance, a total of 171 strains of MDR bacteria were isolated from 95 people, and MDR strains accounted for 49.3% of all isolates. The MDR-GNB bacteria were mainly K. pneumoniae (57.1%), E. coli (11.0%), and Acinetobacter baumannii (8.4%). Of the strains of K. pneumoniae, CRKP accounted for 75.7% and ESBL-producing bacteria accounted for 9.7%. MDR strains accounted for 68.4% of the isolates of A. baumannii and 60.7% of those of E. coli. E. faecium (71.4%) was the main MDR gram-positive bacteria, and MDR strains accounted for 58.8% of the isolates of E. faecium.

### Analysis of Mortality and the Factors Related to the Mortality of the Recipients After KT

Of the 150 patients with early infections, 23 died within one year after transplantation. A total of 69.6% died of infectious complications, 17.4% died of vascular complications, 8.7% died of neurological complications and 4.3% died of other types of complications. Approximately 15.3% of the patients with early postoperative infections died. The overall mean mortality rate after transplantation was 5.5%. Univariate logistic regression analysis showed that the potential risk factors for mortality within 1 year after transplantation were deceased donors, pneumonia, BSI, DGF, MV >48 h, multifocal infection, MDR bacterial infection, CRKP infection, leukopenia after transplantation, reintervention after transplantation and the length of ICU stay. Multivariate logistic regression analysis showed that MV >48 h (OR=13.879, 95% CI: 2.265~85.035; P=0.004) and CRKP infection (OR=6.751, 95% CI: 1.051~43.369; P=0.044) were independent risk factors for mortality within 1 year after surgery, as shown in **Table 1**.

**Table 1:** Univariate and multivariate analysis of risk factors for one-year mortality in 150 kidney transplant recipients with early infection.

Variable	Kidney transplant		Univariate analysis		Multivariate analysis	
	Death (N=23)	Survival (N=127)	OR (95%CI)	P value	OR (95%CI)	P value
Mean age (years)	41.39±8.93	39.02±9.93	1.025 (0.979-1.074)	0.285		
Male, Sex, n (%)	15 (65.22)	93 (73.23)	1.459 (0.568-3.748)	0.433		
Deceased donors, n (%)	**21 (91.30)**	**83 (65.35)**	**5.566 (1.247-24.840)**	**0.024**	1.551 (0.223-10.777)	0.657
Diabetes mellitus, n (%)	8 (34.78)	30 (23.62)	1.724 (0.666-4.462)	0.261		
**Etiology of renal failure**, n (%)						
DM	5 (21.74)	15 (11.81)	2.074 (0.671-6.407)	0.205		
HTA	5 (21.74)	30 (23.62)	0.898 (0.307-2.624)	0.844		
GD	7 (30.43)	40 (31.50)	0.952 (0.363-2.495)	0.920		
Others	6 (26.09)	42 (33.07)	0.714 (0.262-1.944)	0.510		
Acute rejection, n (%)	11 (47.83)	58 (45.67)	1.091 (0.448-2.654)	0.849		
**Types of infection**, n (%)						
Pneumonia	**19 (82.61)**	**48 (37.80)**	**7.818 (2.509-24.354)**	**<0.001**	2.086 (0.343-12.678)	0.425
SSIs	9 (39.13)	47 (37.00)	1.094 (0.440-2.723)	0.846		
BSIs	**11 (47.83)**	**30 (23.62)**	**2.964 (1.187-7.399)**	**0.020**	3.867 (0.632-23.655)	0.143
UTIs	11 (47.83)	51 (40.16)	1.366 (0.560-3.332)	0.493		
DGF, n (%)	**14 (60.87)**	**33 (25.98)**	**4.431 (1.754-11.192)**	**0.002**	1.962 (0.408-9.421)	0.400
ATG induction, n (%)	7 (30.43)	25 (16.69)	1.785 (0.663-4.804)	0.251		
MV>48h, n (%)	**18 (78.26)**	**10 (7.87)**	**42.120 (12.908-137.439)**	**<0.001**	**13.879 (2.265-85.035)**	**0.004**
Multiple infections^a^, n (%)	6 (26.09)	30 (23.62)	1.141 (0.413-3.155)	0.799		
Fungal infections, n (%)	6 (26.09)	29 (22.83)	1.193 (0.431-3.303)	0.735		
CMV, n (%)	6 (26.09)	35 (27.56)	0.928 (0.338-2.544)	0.884		
Multifocal infections^b^, n (%)	**19 (82.61)**	**36 (28.35)**	**12.007 (3.820-37.738)**	**<0.001**	1.182 (0.195-7.169)	0.856
Mixed infectionsc^c^, n (%)	5 (21.74)	23 (18.11)	1.256 (0.423-3.732)	0.683		
Multidrug resistant bacteria infections, n (%)	**20 (86.96)**	**75 (59.06)**	**4.622 (1.306-16.360)**	**0.018**	0.232 (0.022-2.395)	0.220
CRKP infection, n (%)	**16 (69.57)**	**27 (21.26)**	**8.466 (3.162-22.662)**	**<0.001**	**6.751 (1.051-43.369)**	**0.044**
Leukopenia after KT, n (%)	**11 (47.83)**	**8 (6.30)**	**13.635 (4.598-40.438)**	**<0.001**	1.238 (0.203-7.548)	0.817
Postoperative reintervention, n (%)	**18 (78.26)**	**34 (26.77)**	**9.847 (3.392-28.588)**	**<0.001**	2.817 (0.591-13.429)	0.194
Length of ICU stay (days)	**16.96±19.95**	**3.18±7.61**	**1.114 (1.051-1.182)**	**<0.001**	0.995 (0.951-1.041)	0.827

DM, diabetes mellitus; HTA, hypertension; GD, glomerular disease; SSIs, surgical site infections; BSIs, bloodstream infections; UTIs, urinary tract infections; DGF, delayed graft function; ATG, anti-thymocyte globulin MV, mechanical ventilation; ^a^Multiple infections, infections caused by both Gram-negative and Gram-positive bacteria; ^b^Multifocal infection, ≥2sites (blood and urine; blood and pneumonia sites etc. ) were contemporarily involved; ^c^Mixed infections, infections caused by both bacterial and fungal pathogens.

Bold values indicated that these variables were significant in univariate and multivariate analysis (P < 0.05).

### Analysis of Risk Factors for CRKP Infection After KT

**Figure 2:**
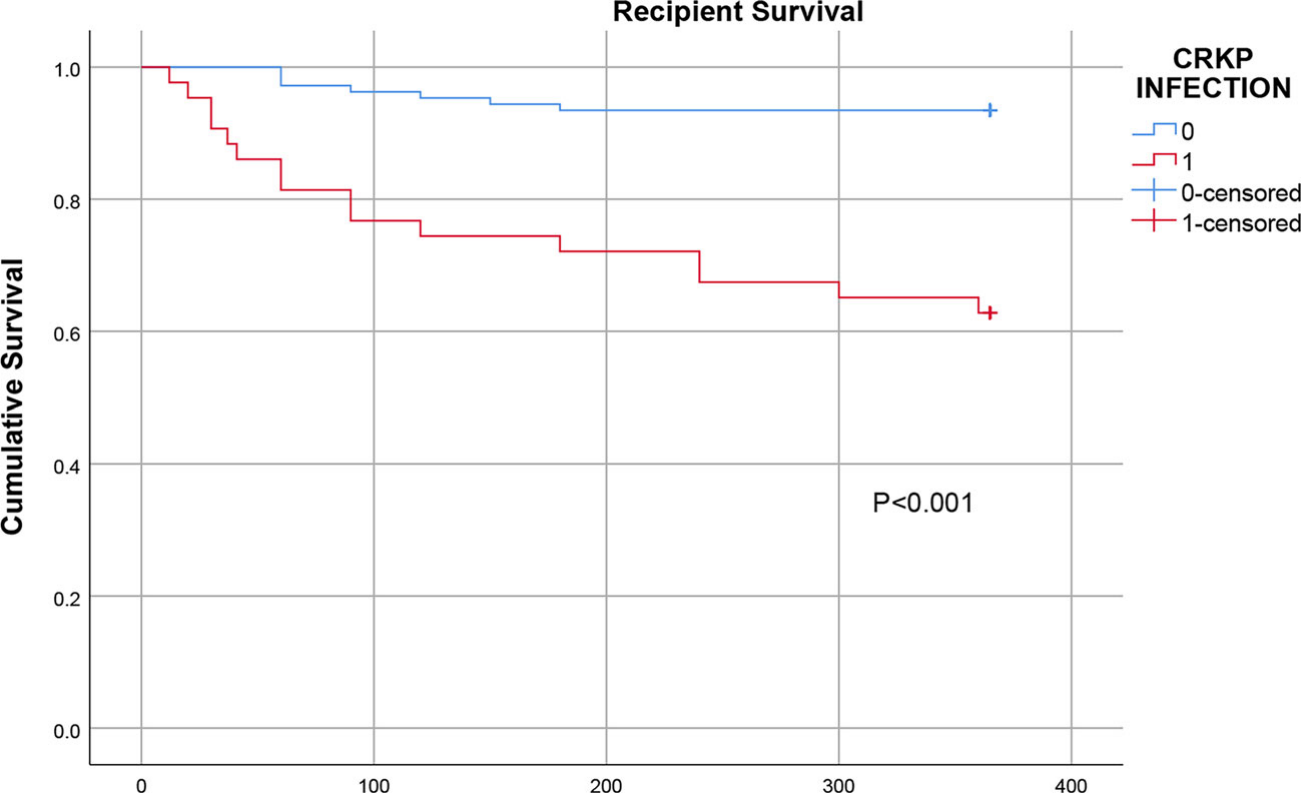
Comparison of the one-year survival rate of infected and uninfected CRKP among early KT recipients.

Of the 415 KT recipients, 43 patients, accounting for 10.4% of the total population, were infected with CRKP, and the rate of CRKP infection-related mortality was 37.2% (16/43). Of the surviving recipients, 6 underwent graft nephrectomy due to CRKP infection. The median time from transplantation to the first CRKP infection was 28 days (IQR 17-40 days), In terms of CRKP infection sites, there were 25 cases of pneumonia, 23 cases of SSIs, 15 cases of BSIs and 13 cases of UTIs. 32 (74.4%) of the 43 patients with CRKP infection received tigecycline based antibiotic regimen, 8 (18.6%) received aminoglycosides based antibiotic regimen, and 3 (7.0%) received ceftazidime- avibatan or Polymyxin antibiotic regimen. Univariate logistic regression analysis showed that the potential risk factors for CRKP infection were DGF, leukopenia after KT, reintervention after transplantation, MV >48 h and the length of ICU stay. Multivariate logistic regression analysis showed that MV >48 h (OR=3.719, 95% CI: 1.024-13.504; P=0.046) was an independent risk factor for CRKP infection, as shown in **Table 2**. The antibiotic sensitivity of CRKP isolates are shown in **Table 3**. According to Kaplan-Meier analysis, the overall survival rate of CRKP-infected patients was significantly lower than that of non-CRKP-infected patients, as shown in **Figure 2**. (P<0.001).

**Table 2:** Univariate and multivariate analysis of the risk of carbapenem-resistant Klebsiella pneumoniae infection in 150 kidney transplant recipients.

	CRKP infection		Univariate analysis		Multivariate analysis	
Variable	Yes (N=43)	No (N=107)	OR (95%CI)	P value	OR (95%CI)	P value
Mean age (years)	**42.30±7.92**	**38.21±10.25**	**1.045 (1.006-1.086)**	**0.022**	1.030 (0.986-1.076)	0.181
Male, Sex, n (%)	32 (74.42)	76 (71.03)	0.843 (0.378-1.880)	0.676		
Deceased donors, n (%)	**35 (81.40)**	**69 (64.49)**	**2.409 (1.015-5.717)**	**0.046**	1.206 (0.442-3.292)	0.715
Diabetes mellitus, n (%)	13 (30.23)	25 (23.36)	1.421 (0.645-3.132)	0.383		
**Etiology of renal failure, n (%)**						
DM	7 (16.28)	13 (12.15)	1.406 (0.519-3.806)	0.502		
HTA	9 (20.93)	26 (24.30)	0.825 (0.350-1.944)	0.659		
GD	14 (32.56)	33 (30.84)	1.083 (0.507-2.311)	0.838		
Others	13 (30.23)	35 (32.71)	0.891 (0.414-1.917)	0.769		
Acute rejection, n (%)	22 (51.16)	47 (43.93)	1.337 (0.658-2.719)	0.422		
DGF, n (%)	**19 (44.19)**	**28 (26.17)**	**2.234 (1.065-4.683)**	**0.033**	1.196 (0.468-3.060)	0.708
ATG induction, n (%)	7 (16.28)	25 (23.36)	0.638 (0.253-1.609)	0.341		
MV>48h, n (%)	**18 (41.86)**	**10 (9.34)**	**6.984 (2.870-16.995)**	**<0.001**	**3.719 (1.024-13.504)**	**0.046**
CMV, n (%)	8 (18.60)	33 (30.84)	0.513 (0.215-1.224)	0.132		
Leukopenia after KT, n (%)	**13 (30.23)**	**6 (5.61)**	**7.294 (2.553-20.838)**	**<0.001**	2.721 (0.737-10.050)	0.133
Postoperative reintervention, n (%)	**21 (48.84)**	**31 (28.97)**	**2.340 (1.128-4.853)**	**0.022**	0.903 (0.334-2.437)	0.840
Length of ICU stay (days)	**10.19±19.34**	**3.33±4.94**	**1.064 (1.015-1.114)**	**0.009**	1.005 (0.961-1.052)	0.825

DM, diabetes mellitus; HTA, hypertension; GD, glomerular disease; DGF, delayed graft function; ATG, anti-thymocyte globulin; MV, mechanical ventilation.

Bold values indicated that these variables were significant in univariate and multivariate analysis (P < 0.05).

**Table 3:** Antimicrobial susceptibility of isolates from patients with carbapenem-resistant klebsiella pneumoniae infection.

Antibiotic	Number of isolates tested (N)	Susceptible (%)
Ceftazidime	43	0.0%
Levofloxacin	43	9.3%
Gentamycin	40	12.5%
Aztreonam	43	0.0%
Imipenem	43	0.0%
Meropenem	43	0.0%
Amikacin	40	20.0%
Polymyxin	42	97.6%
Tigecycline	43	100.0%

## Discussion

Due to their poor physical function, frequent need for antibacterial treatment, multiple hospitalizations, and immunosuppression, SOT recipients have a higher risk of infection with MDR bacteria (Barchiesi et al., 2016; Pouch and Satlin, 2017). In our study, we investigated the prevalence of early bacterial and fungal infections and the distribution of pathogens after KT. Of the 415 recipients, 150 (36.1%) had at least one infection recorded within 90 days after transplantation, of whom 23 died within a year after transplantation. The overall infection rate was 36.1%. To date, no study has reported the overall prevalence of early infections after KT. The overall postoperative infection rate in our study was higher than that after liver transplantation, which Barchiesi et al (Barchiesi et al., 2016) reported was 26.7%, possibly due to the inclusion of donor-derived infections in our study. Although we investigated 20 demographic, clinical and microbiological characteristics, only two factors were independently associated with 1-year mortality, namely, MV> 48 h and CRKP infection. A possible explanation for this finding is that patients requiring MV are more likely to use invasive devices and be admitted to intensive care units, both of which increase the risk of CRKP infection (Patel et al., 2008; Giannella et al., 2015). Pneumonia and BSIs were identified in univariate analysis, but they did not remain significant in multivariate analysis, indicating that the type of infection had no effect on the results.

In our study, CRKP infection was found to be an independent risk factor for one-year mortality after KT. The same conclusion was reached in two recent studies on liver transplantation. Those two studies showed that when liver transplant recipients were infected with CRKP, the 1-year survival rate decreased significantly from 86% to 29% and from 93% to 55% (Kalpoe et al., 2012; Pereira et al., 2015). In this study, a total of 43 recipients had CRKP infections, yielding an infection rate of 10.4%. In terms of the prevalence, published studies have reported different CRKP infection rates ranging from 3% to 11.2% (Clancy et al., 2013; Varotti et al., 2017; Bias et al., 2018). In our study, it was found that the prevalence of CRKP infection was very high, possibly because our institute is located in an area with a high incidence of CRKP infection. CHINET data show that the drug resistance rates of K. pneumoniae to imipenem and meropenem increased from 2.9% to 23.7% and 3% to 25%in China from 2005 to 2019, respectively, and the incidence of CRKP infection has also increased sharply worldwide in the past decade (, 2018).

Of the 43 patients with CRKP infections, 16 (37.2%) died. Previous studies showed that the overall mortality of transplant recipients with CRKP infections was as high as 40%~75% (Mazza et al., 2017; Bias et al., 2018; Wang et al., 2020). In our study, the Kaplan-Meier curves showed that the one-year survival rate after transplantation in the CRKP-infected group was significantly lower than that in the control group. This confirms previous the data reported in in the literature, which generally suggest that CRKP infection has a negative effect on outcomes in renal transplant patients. The resistance of CRKP to most antibiotics is an important factor affecting the high mortality rate. In addition, the toxicity and side effects of antibiotics, immunocompromise, graft insufficiency requiring hemodialysis and mixed infections are also important reasons for the high mortality rate. Some studies have reported that allograft nephrectomy can improve the antimicrobial treatment success rate (Clancy et al., 2013; Simkins et al., 2014). In our study, 6 of the surviving patients underwent graft nephrectomy, complete debridement and drainage, which shows that drainage, debridement and other mechanical methods of removing infected lesions are important auxiliary means of managing CRKP infections.

Other studies have also predicted risk factors for CRKP infections, such as DGF, diabetes (Varotti et al., 2017; Taminato et al., 2019; Vigara et al., 2020). In our analysis, these factors were not associated with a higher risk of CRKP infection. Instead, MV > 48 h was found to be an independent risk factor for CRKP infection, which is consistent with the results obtained in previous studies (Patel et al., 2008; Giannella et al., 2015). The graft loss rate and mortality rate in the control group we selected were higher than the average rates at the transplant center, and their condition was more severe than that of the recipients without any infections. We did not select KT recipients who did not have infections as the control group to facilitate the identification of the factors associated with CRKP infection. In the analysis of the risk factors for CRKP infection, donor-derived infections have been reported as a mechanism of transmission (Mularoni et al., 2015; Varotti et al., 2016).

This study has several limitations. Firstly, the results of this retrospective study, which only included patients from a single institute, may not be applicable to KT recipients in other institutions or at other locations. Secondly, in the analysis of risk factors for CRKP infection, various studies have emphasized the importance of the colonization status of patients before and after transplantation (Giannella et al., 2015; Mazza et al., 2017). Unfortunately, we have not been able to collect these data owing to the absence of routine monitoring and screening. Thirdly, we did not carry out gene identification and drug resistance mechanism detection of CRKP strain.

## Conclusion

In summary, our research showed that the prevalence of CRKP infection is higher in KT recipients. Patients who undergo MV> 48 h are more likely to develop CRKP infections. CRKP infections negatively affect the prognosis of KT recipients and significantly reduces their survival. Therefore, appropriate isolation and intervention should be performed for high-risk patients to prevent the spread of drug-resistant bacteria.

## Data Availability Statement

The datasets used and/or analysed during the current study are available from the corresponding author on reasonable request.

## Ethics Statement

The studies involving human participants were reviewed and approved by the ethics committee of The First Affiliated Hospital of Anhui Medical University. Written informed consent for participation was not required for this study in accordance with the national legislation and the institutional requirements. Written informed consent was not obtained from the individual(s) for the publication of any potentially identifiable images or data included in this article.

## Author Contributions

FZ, JZ, and HD: study design, statistical analysis, data interpretation, manuscript preparation, literature search. JP: data collection. JY: data collection. TL: data collection. YC: data collection. GL: study design, provision of materials and resources, data interpretation. All authors contributed to the article and approved the submitted version.

## Funding

This work received funding from the Natural Science Foundation of Anhui Province (Grant No. 1508085SMH226).

## Conflict of Interest

The authors declare that the research was conducted in the absence of any commercial or financial relationships that could be construed as a potential conflict of interest.

## Publisher’s Note

All claims expressed in this article are solely those of the authors and do not necessarily represent those of their affiliated organizations, or those of the publisher, the editors and the reviewers. Any product that may be evaluated in this article, or claim that may be made by its manufacturer, is not guaranteed or endorsed by the publisher.

## Abbreviations

BSIs, bloodstream infections**;** CMV, cytomegalovirus**;** CRKP, carbapenem-resistant Klebsiella pneumoniae**;** DGF, delayed graft function; ICU, intensive care unit; KT, kidney transplant**;** MV, mechanical ventilation**;** MDR, multidrug-resistant**;** MDR-GNB, multidrug-resistant gram-negative bacteria**;** SOT, Solid organ transplant**;** SSIs, surgical site infections**;** UTIs, urinary tract infections.
